# Mature Cardiac Teratoma in an Adult

**DOI:** 10.4021/cr182w

**Published:** 2012-05-20

**Authors:** Ronny A Cohen, Pablo Loarte, Victor Navarro, Brooks Mirrer

**Affiliations:** a NYU School of Medicine, New York, NY, USA; b Woodhull Medical Center, Brooklyn, NY, USA

**Keywords:** Mature Teratoma, Treatment, Diagnosis

## Abstract

The incidental diagnosis in adult age is very unusual and the presence of clinical symptoms is related to its location, which is most commonly intrapericardial. The presence of intramyocardial teratoma lesions is even rarer and has been reported in few publications. The recommendations for the diagnosis and management of a cardiac teratoma depends upon the imaging studies and the pathological report after surgical excision. The prognosis of surgically treated patient is very good and a complete surgical excision is preferred in order to avoid complications.

## Introduction

Teratomas are tumors of embryonic origin composed of elements derived from the three germinal layers in varying degrees [1]. If more than 50 percent of the tumor is comprised of well-differentiated elements, then the tumor is referred to as a mature teratoma.

The most frequent site of teratomas is the gonads followed by the mediastinum [1, 2]. Ninety percent of the cardiac teratomas have been found in the pericardium and the rest in the myocardium [3].

## Cardiac Teratoma

Their occurrence in adults is very rare (less than 1%) [4, 5].

The peak incidence at presentation is in the second and third decades of life. There is a slight female sex predominance among the reported case in adult population. A simpler form of “teratoma”, for which the distinction has been blurred, is the teratodermoid or dermoid cyst, composed predominantly of derivatives of epithelial layers, including dermal and dermal glands, hair and sebaceous material. Teratomas are histologically more complex, composed of three embryonic layer-orgin cell types. The solid components of the so-called “mature” cystic teratoma may contain well differentiated elements of bones, cartilage, teeth, muscle, connective tissue, fibrous and lymphoid tissue, nerve, thymus, mucous and salivary glands, lung, liver and pancreas. The mature cystic teratoma is generally benign, though both immature and mature types may show malignant transformation, with diagnostic aggressive growth characteristics on histologic examination, resembling a non-GCT somatic-type cancer. Additionally, in categorizing malignant germ cell tumors, alpha feto protein and HCG evaluation are useful in differentiating seminomas from non-seminomatous tumors, assessing response to therapy in hormonally active tumors and diagnosing relapse or failure of therapy [3, 6].

Pathologically, these tumors are multicystic with a size that goes from few millimeters to several centimeters up to 15 cm. They have a smooth surface and are lobulated. When tumors are large, can cause pericardial effusion [3, 7].

Among all the cardiac tumors, teratomas are grouped within the primary benign tumors, which account for 7% of cardiac tumors. The majority of the teratomas are located in the pericardium and can produce constrictive pericarditis [8]. The benign tumors include myxomas, lipomas, fibroelastomas, rhabdomyomas, hamartomas and others more. The malignant teratomas are included apart among the primary malignant tumors of the heart [9].

The anterior mediastinal compartment (also known as anterosuperior compartment) is anterior to the pericardium and includes lymphatic tissue, the thymus, the extrapericardial aorta and its branches, and the great veins [1]. Mediastinal masses are generally located in the anterior mediastinum and more often tend to be malignant in nature. The tumors most frequently seen in this region are thymomas (32%), lymphomas (23%) and germ cell tumors (17%).

Germ cell tumors are thought to originate from primordial germ cells that fail to complete migration from the urogenital ridge and come to rest in the mediastinum. They are classified into teratomas, teratocarcinomas, seminomas, embryonal cell carcinomas, choriocarcinomas, and endodermal cell (yolk sac) tumors [1].

Cabanas and Moore reported in 1973 the first malignant intracardiac teratoma. The tumor filled partly the right ventricle consisting of various mature and immature elements. Sarcomatous tissue consistent with leimyosarcoma, stratified keratinizing squamous epithelium, and poorly differentiated small epithelial cells comprised the malignant components. These tumors have the propensity to originate from the interatrial and interventricular septum and grow into the right chambers. Metastasis although rare can be to the lungs, brain and bones [6].

As mentioned above, teratomas are mostly found in the pericardium and along with malignant mesotheliomas, are the most common pericardial tumors. The pericardial teratomas are usually right-sided masses, usually connected to one of the great vessels via a pedicle. Most of them lie within the pericardial sac and rarely can be intramyocardial. The intramyocardial lesions have occurred in newborns or in the first 6 years of life and most of them are asymptomatic but heart failure and sudden death may occur. Death from arrhythmia is caused by intraventricular location [5, 7].

## Symptoms of Mature Teratomas of the Mediastinum

The usually benign mature cystic teratomas of the mediastinum often grow slowly: as a result, they are more likely to be diagnosed incidentally while they are still asymptomatic. Symptoms when present are related to mechanical effects and include chest pain, cough, dyspnea, bronchial obstruction with postobstructive pneumonia, and rarely palpitations. Erosion into an adjacent bronchus can rarely lead to expectoration of hair (trichoptysis) or sebaceous debris, a finding pathognomonic of benign teratoma. The presence of pericardial effusion with compression is usually due to rupture rather than to the size of the tumor itself. Anterior mediastinal teratoma causing cardiac tamponade is a rare entity [10, 11]. Also, there have been few documented cases of mediastinal teratomas producing significant amount of hormones (insulin, glucagon and somatostatin).

## Diagnosis

**Figure 1:**
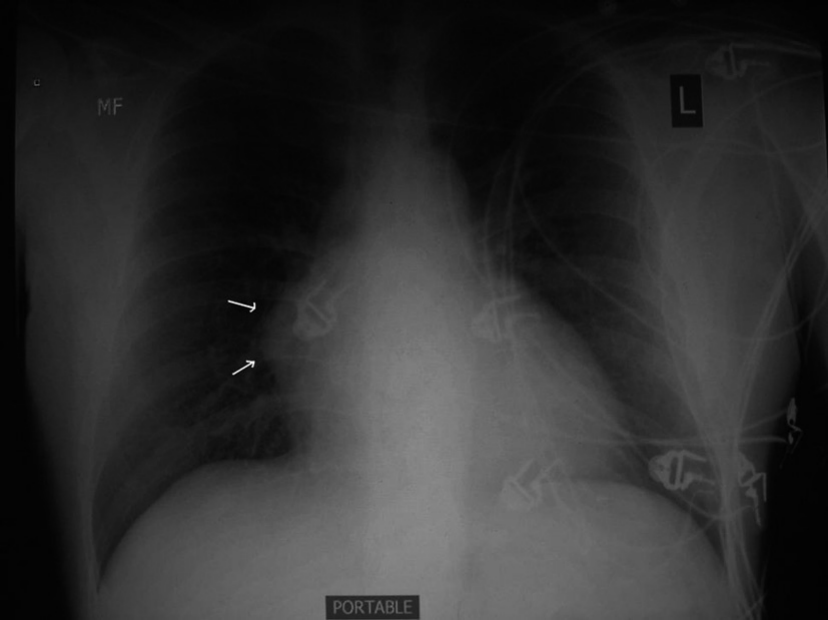
Chest radiograph (postero-anterior view) with calcification in the right heart border (arrows).

**Figure 2:**
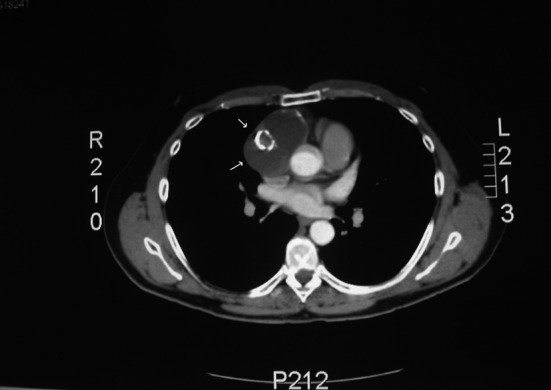
Chest computed tomography (axial view) with cystic lesion in the pericardium (arrows).

Chest radiography, if performed incidentally or for symptoms, typically shows an anterior mediastinal mass, with calcification seen in 25 percent of benign teratomas and an enlarged cardiomediastinal silhouette (Fig. 1) [1, 2, 7]. In some patients, well-formed teeth or bone are seen on plain film, findings that are very suggestive of the diagnosis. CT and MRI are helpful in localizing lesions and determining the spatial relationships to surrounding structures. They can also characterize densities within the lesion suggestive of fat, sebaceous material, or cystic elements. CT is a good initial test (Fig. 2), but in certain studies MRI has been considered mandatory because it determines better the pericardial involvement as mentioned above, it relationship with the surrounding structures such as vessels, pericardium or myocardium [5, 11, 12].

**Figure 3:**
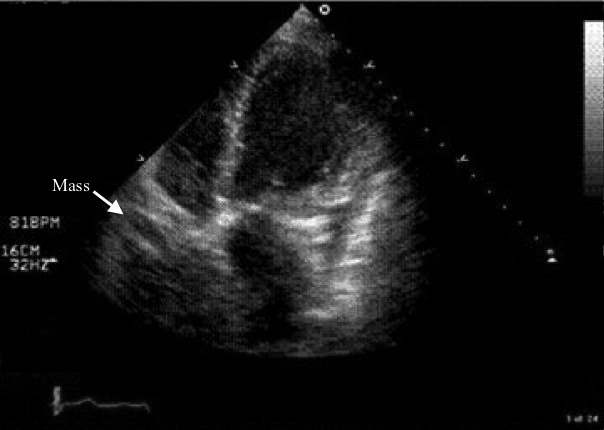
Transthoracic 4 chamber echocardiogram poorly defined pericardial mass overlying right atrial border (arrow) [18]

Echocardiogram characterizes an intrapericardial mass and complex multilocular cyst mostly in the right side of the heart (Fig. 3). Associated pericardial effusion can be found, attachment to aorta through a pedicle and intrinsic echogenic focis representing calcifications are other findings [4].

The diagnosis is always made by pathologic evaluation after surgical excision. The transthoracic needle biopsy is still on debate because there are possible serious complications with a low yield of tissue for accurate diagnosis and their use should be well discussed and considered only for selected case [5].

In general the differential diagnosis includes fibromas, rhamdomyomas and hemangiomas.

## Treatment and Prognosis

Mature teratomas are relatively insensitive to both chemotherapy and radiation therapy. Treatment of mediastinal mature teratomas is surgical excision and this is almost always curative. Beck was the first to successfully resect the tumor from a patient in 1938 [13]. Deenadayalu et al. documented the youngest patient, a two-week-old female, successfully treated through surgery [14]. Subtotal resection with relief of compressive symptoms is performed if benign teratomas cannot be excised completely without endangering surrounding vital structures. The complete resection is preferred in order to avoid pericardial effusions and hemodynamic compromise derived from the later and related to remaining tissue from incomplete resection. Resection generally is through a median sternotomy or posterolateral thoracotomy, depending upon the location of the tumor, although thoracoscopic resection is occasionally possible. The prognosis of surgically treated patients is good [5, 7, 15-17].

These tumors should be excised whenever detected because they 1.) Carry a risk for malignant transformation, 2.) May lead to compression and arrhythmias due to its proximity to surrounding vital structures and 3.) May carry the likelihood of becoming infected. Surgical excision usually does not pose much problem, as very often these tumors are pedunculated. However, since the vascular supply is derived from the adventitial vessels of the aorta, there is a small risk of massive hemorrhage from the aorta during dissection.
